# Glucagon-Like Peptide-1 Receptor Agonists (GLP-1RAs) for Obesity and Symptoms in Menopause: A Review

**DOI:** 10.7759/cureus.101693

**Published:** 2026-01-16

**Authors:** Nicole A Graczyk, Julia Bisschops

**Affiliations:** 1 Medical Education, Herbert Wertheim College of Medicine, Miami, USA; 2 Family Medicine, Herbert Wertheim College of Medicine, Miami, USA

**Keywords:** glp-1 receptor agonist, hormone replacement therapy, menopause, obesity, postmenopausal women, vasomotor symptoms, weight management

## Abstract

Background: The effects of glucagon-like peptide-1 receptor agonists (GLP-1RAs) in menopausal and postmenopausal women are not well characterized and may differ from other patient populations, given their different hormone profiles. Current research evaluating the effects of GLP-1RAs on weight loss, cardiovascular markers such as total cholesterol, and vasomotor symptoms (VMS) in this patient population is limited.

Objective: This scoping review summarizes current articles on the effects of GLP-1RAs on central adiposity, VMS, and cardiovascular markers in menopausal and postmenopausal women.

Methods: A scoping search of PubMed and Embase was conducted to identify papers that explore the role of GLP-1RAs in menopause. Articles were chosen based on title and abstract relevance, study design, and metabolic variables used.

Results: Across the selected studies, GLP-1RAs were associated with increased weight loss and a decrease in central adiposity in menopausal and postmenopausal women. Limited studies also reported improved VMS and cardiovascular markers; however, further research is needed.

Conclusions: GLP-1RAs may offer benefits for menopausal women, especially regarding weight gain and VMS. Larger, more robust studies conducted in menopausal women are needed to determine effects on factors such as cardiovascular markers and bone density.

## Introduction and background

Menopause, which typically occurs around the age of 51, though the age of onset may vary by geography and ethnicity, marks the transition to a hypoestrogenic state in women [1]. In the months to years leading up to this stage, rising levels of follicular-stimulating hormone and luteinizing hormone reflect the body’s attempts to stimulate ovarian estrogen production. However, as ovarian function declines, this feedback loop becomes ineffective, resulting in menopause. This transition is often accompanied by an increased risk of cardiovascular disease (CVD), vasomotor symptoms (VMS), and an increase in central adiposity. The latter is particularly concerning, as 60%-70% will experience an increase in weight and central obesity during this time [2]. In a population-based study conducted by Woolcott et al., it was found that 56.4% of reproductive-age women and over 75% of postmenopausal women were obese, providing evidence that there is a significant increase in weight following this transition [3]. Therefore, management of weight gain in menopausal women is of significant importance in the field of women’s health.

Evidence suggests that weight gain associated with menopause increases the risk of CVD, independent of other variables commonly attributed to CVD [4]. While studies of different methodologies, including Mendelian randomization analyses, report mixed results, clinical evidence supports an association between menopause-related sex hormone changes and increased CVD risk [5]. These findings suggest the need for well-designed longitudinal observational studies to characterize underlying mechanisms of CVD risk in this patient population, as well as randomized control trials (RCTs) of therapeutics to ascertain their benefit in menopausal women. 

A variety of interventions currently exist to manage the sequelae of changes that occur with weight gain in menopause, including lifestyle modifications, hormonal replacement therapy (HRT), pharmacologic agents, and bariatric surgery. Recently, glucagon-like peptide-1 receptor agonists (GLP-1RAs) have gained popularity as a therapeutic agent for weight loss in patients with various weight-related conditions. In large clinical trials, GLP-1RAs have been shown to produce significant reductions in weight in overweight or obese participants with and without diabetes [6]. Additionally, multiple trials demonstrate that patients who are obese or overweight treated with GLP-1RAs had decreased metabolic risk factors such as total cholesterol, triglycerides, systolic and diastolic blood pressure, and BMI [7]. Such evidence supports the potential benefit of GLP-1RA used in the management of menopause-associated changes in CVD risk and weight gain. 

Despite increasing use of GLP-1RAs for various obesity and metabolic-related conditions, menopausal women remain underrepresented in clinical trials, and few studies evaluate the use of GLP-1RAs in the management of increased central adiposity, VMS, CVS risk, and bone health. This review examines the potential implications for the use of GLP-1RAs in the management of metabolic changes during the menopausal transition, specifically changes in central adiposity, VMS, and cardiovascular risk factors. Preliminary findings from this review were presented as a poster at the 2025 Nova Southeastern Women’s Health Conference in Davie, FL, August 2025.

## Review

Methods

A PubMed search for abstracts and full articles was conducted using the following MeSH terms: (menopause[Text Word] OR menopausal[Text Word] OR postmenopause[Text Word] OR postmenopausal[Text Word] OR perimenopause[Text Word] OR perimenopausal[Text Word] OR climacteric[Text Word]) AND (glp[Text Word] OR glp1[Text Word] OR glucagon like peptide[Text Word] OR rglp[Text Word] OR semaglutide[Text Word] OR dulaglutide[Text Word] OR liraglutide[Text Word] OR exenatide[Text Word] OR tirzepatide[Text Word]). 

This search yielded 114 results. A similar search was conducted on Embase, but was expanded to include conference presentation posters.

Titles and abstracts were screened for relevance to menopausal status and GLP-1-based therapies. Full-text articles were reviewed when available. Studies were included if they evaluated outcomes related to weight, central adiposity, VMS, cardiometabolic markers, or bone health in perimenopausal or postmenopausal women, or in estrogen-deficient models when human data were limited. Given heterogeneity in study design, populations, and outcome reporting, findings were synthesized descriptively, and no formal risk-of-bias assessment or quantitative meta-analysis was performed.

Metabolic changes in menopause

The increase in body weight observed in women from a premenopausal to postmenopausal state is somewhat age-dependent, as women, on average, gain 0.5 kg yearly, regardless of reproductive status [8]. A review conducted by Moccia et al. explored the potential relationship between hormones and the weight gain observed during menopause [9]. They assert that multiple mechanisms likely contribute to the accumulation of central adipose tissue, such as a decrease in estrogen and an increase in androgens [9]. However, hormonal factors likely contribute to where the adipose is distributed.

Supporting this hypothesis, both rodent and human studies have demonstrated an association between estrogen decline and an increase in visceral fat. In ovariectomized rats, there was an increase in visceral fat, BMI, and body weight compared to the control group [10]. Similarly, a study conducted by Shea et al. (2015) found an increase in visceral fat in women when menopause was induced via gonadotropin-releasing hormone unless they were also treated with estradiol [11]. Of note, while this RCT did not find a significant change in overall body weight, it does demonstrate a shift in fat distribution. Finally, another study analyzing the glycemic effect of HRT in postmenopausal women found a decrease in weight and BMI [12]. These findings suggest estrogen plays a role in the shift of fat distribution in menopause, but other factors likely contribute to the overall weight gain associated with this transition.

The loss of estrogen in menopause also contributes to the development of osteoporosis in this population. An insufficient level of estrogen results in increased bone resorption due to stimulation of osteoclasts. As these cells resorb bone, osteoblast function is decreasing (by mechanisms that are not yet fully understood) [13]. These processes result in a net loss of bone that predisposes menopausal women to osteoporotic fractures, which can often have devastating effects. Therefore, therapeutic treatment of menopause should also include therapies to mitigate the potential harmful effects of this condition.

Metabolic Syndrome

Metabolic syndrome (MetS) is a multisystem condition characterized by the presence of three or more of the following: hyperlipidemia, hypertension, low high-density lipoprotein (HDL) levels, increased central adiposity, or insulin resistance. Together, these factors contribute to an increased risk of developing CVD and type 2 diabetes.

During the perimenopausal and early postmenopausal periods, the prevalence of MetS increases significantly compared with premenopausal women [14]. As of 2016, 50% of women over the age of 60 are affected by MetS [15]. One proposed mechanism of this increased risk is the decline in estrogen, as this hormone has been found to decrease levels of low-density lipoprotein (LDL) [16]. However, there has been conflicting data regarding the change in HDL observed in postmenopausal women. Therefore, research is needed to further elucidate the role of estrogen and the changes observed in cholesterol trends in menopause.

Estrogen has also been suggested to modify disease risk factors by regulating the formation of damaging molecules. Typically, estrogen functions to increase the production of antioxidant molecules, helping to decrease oxidative stress. A study conducted by Kim et al. found increased levels of oxidized LDL in postmenopausal women, suggesting a possible mechanism for the increased risk of CVD in this patient population [17]. This molecule is an important mediator of atherosclerotic heart disease, another component of MetS. These findings highlight the importance of weight management during this hormonal transition period of menopause.

Current strategies for managing menopausal weight gain and symptoms

Lifestyle Intervention

Early interventions for managing weight gain during menopause include changes in nutrition and lifestyle. These recommendations follow the national nutritional guidelines--four to five portions of vegetables a day, reduce calorie intake to avoid the weight gain that is typical in old age, and increase fiber intake [2]. While these recommendations are important for all women transitioning to a postmenopausal state, they are particularly important for those with other metabolic disorders, such as type 2 diabetes and CVD. For these women, recommendations are focused on limited carbohydrate intake and natural sources of these macromolecules when possible. Another approach is that of the Mediterranean diet, which emphasizes the consumption of antioxidant foods with anti-inflammatory effects in the body [18]. Incorporating these micronutrients through foods such as fruits and leafy greens can also contribute to improved cardiovascular health and insulin resistance [18]. 

Interestingly, a study conducted in 2012 found that changes in diet and weight loss both decreased the risk for the development of VMS, i.e., hot flashes. Researchers found that women who lost ≥10% of their initial weight had a 56% higher likelihood of not experiencing hot flashes (odds ratio (OR): 1.56, 95% CI: 1.21-2.02). In contrast, those who gained weight and had improved their diet by increasing whole grain, fruit, and vegetable intake while reducing fat intake still reduced VMS [19]. These findings suggest that diet and exercise must also be emphasized when counseling patients on the lifestyle modifications to manage symptoms during perimenopause.

Exercise also provides benefits to menopausal women by promoting bone density and managing associated symptoms of menopause. A randomized controlled trial conducted by Berin et al. found that with 15 weeks of resistance training, postmenopausal women experienced a reduction in the frequency and severity of hot flashes [20]. This effect persisted for six months after the trial, but benefits were lost after two years of follow-up [21]. These findings emphasize the importance of continuing strength training beyond the menopausal transition period. Moreover, resistance training is a well-known form of combating bone density loss in menopause. In a randomized controlled trial conducted by Watson et al., postmenopausal women have increases in spinal lumbar bone mineral density and smaller declines in bone mass in other areas [22]. Therefore, resistance training offers multiple benefits and represents a powerful non-pharmacologic tool for improving quality of life in this population.

Pharmacologic Agents

Pharmacologic agents are used in the management of menopausal symptoms when non-pharmacologic intervention fails to adequately regulate symptoms. These include hormonal therapy replacement, antidepressants, selective estrogen receptor modulators, and recently, GLP-1RAs. Implications for the use of GLP-1RAs will be explained in depth later.

HRT is indicated for women experiencing VMS, i.e., hot flashes and night sweats. Moreover, an RCT investigating the relationship between HRT and diabetes in postmenopausal women found that HRT prevents the normal increase in glucose observed during this time [12]. As such, HRT is regularly indicated for use in women experiencing moderate-to-severe symptoms of menopause. Antidepressants such as paroxetine are also used, as a decrease in estrogen is associated with a decrease in serotonin [23]. Thus, selective serotonin reuptake inhibitors/serotonin-norepinephrine reuptake inhibitors help maintain an adequate level of free serotonin available, improving VMS.

Bariatric Surgery

Weight loss during menopause can also be achieved by bariatric surgery, including gastric bypass and vertical banded gastroplasty [8]. However, the indications for this surgery limit its use, and it is exceedingly rare to address menopausal weight gain with bariatric surgery. Moreover, studies have found that postmenopausal women had lower total body weight loss and smaller changes in BMI following bariatric surgery [24]. Therefore, while bariatric surgery is an effective option, its use may be limited in the treatment of postmenopausal women.

GLP-1RAs: a novel therapeutic approach

Recently, GLP-1RAs have gained popularity for treating chronic weight-related conditions. These medications enhance the sensitivity of beta cells in the pancreas to glucose, thereby lowering blood glucose levels. Glucagon-like peptide-1and GLP-1RAs also suppress gastrointestinal motility and delay gastric emptying, contributing to weight loss. Additionally, they may induce satiety by targeting receptors in the central nervous system [25]. Given the multiple mechanisms by which GLP-1RAs work, research is ongoing to determine the full scope of conditions and symptoms treatable with this class of medications.

Metabolic Effects in Estrogen-Deficient Models

Interactions between GLP-1RAs and lipolysis in estrogen-deficient states are complex. Model et al. suggest that GLP-1RAs and estrogens have opposing effects on white adipose tissue fat and glucose metabolism [26]. For instance, glucagon-like peptide-1 may counteract lipid accumulation typically promoted in estrogen-deficient states, as evidenced by increased basal lipolysis in mice treated with liraglutide (p=0.011) [26]. Additionally, other studies have shown improvements in fasting insulin resistance indices in mice treated with tirzepatide (p<0.001) [27]. These findings support the notion that further research needs to be conducted in human clinical trials to define the potential benefits of GLP-1RAs in the management of menopausal symptoms.

Clinical Efficacy in Postmenopausal Women

Preliminary research indicates that GLP-1RAs are effective in treating obesity in menopausal women. These medications, specifically semaglutide and liraglutide, have demonstrated comparable findings regarding weight loss, lean muscle mass loss, and fat mass loss in both premenopausal and postmenopausal women [28,29]. Such findings suggest that GLP-1RAs are effective in women regardless of menopausal status. Additionally, GLP-1RAs have proven effective in postmenopausal women with a BMI both below and above 35 kg/m² [30]. While this data is preliminary, it asserts that GLP-1RAs can also be effective in more severe cases of obesity among postmenopausal women. Finally, semaglutide may have greater patient compliance, as it is longer-acting and can be administered once weekly instead of once daily, like in liraglutide. However, both are regarded as adequate medications for treating chronic metabolic conditions associated with obesity.

Impact on Central Adiposity

Central adiposity also decreased significantly with GLP-1RA treatment. For instance, a cross-sectional observational study conducted by Tchang et al. reported a 20-cm reduction in waist circumference with tirzepatide in menopausal women, compared with a 4-cm reduction with placebo (p<0.001) [31]. Similarly, Nicolau et al. observed a significant reduction in waist circumference following treatment with semaglutide [29]. Both studies were conducted in obese or overweight women. It is important to note that Nicolau et al. used semaglutide in conjunction with structured lifestyle interventions. Therefore, diet and exercise may have had a confounding effect on the change in waist circumference experienced by the women in this study. Nonetheless, further research is still warranted to determine the role of GLP-1RAs in the prevention of MetS in menopausal women.

Combination Therapy With HRT

Menopausal women may benefit from a combination of hormone therapy (HT) and GLP-1RAs. A retrospective study by Hurtado et al. provides additional support for the use of GLP-1RAs in reducing the risk of MetS among menopausal women [32]. This study found that combining HT with semaglutide improved cardiometabolic risk factors in women compared with those taking semaglutide alone. Specifically, total cholesterol, glycated hemoglobin, and triglyceride counts improved in those receiving both HT and semaglutide, while those receiving only semaglutide did not have improved triglyceride and total cholesterol levels [32]. Similarly, preliminary real-world findings presented at the 2025 Endocrine Society annual meeting reported that women who received tirzepatide combined with HT experienced a greater total body fat loss compared with those using tirzepatide alone [33]. These findings suggest that there may be a) a synergistic effect between HT and GLP-1RAs or 2) different mechanisms contributing to the improvement of risk factors in those receiving both treatments. Further research is warranted to clarify the metabolic processes at play to appropriately use GLP-1RAs in this patient subpopulation.

VMS and Quality of Life

Many of the symptoms experienced by menopausal women have been shown to be at least somewhat correlated with weight gain. For example, research has shown that weight loss lessens and/or delays the onset of VMS in menopausal women [2]. Ongoing research suggests that GLP-1RAs may be useful in managing hot flashes in menopausal women, with these VMS potentially serving as a proxy for cardiovascular health [30]. Therefore, the use of GLP-1RAs in menopausal women may alleviate multiple symptoms associated with this hormonal transition by limiting weight gain during this time.

These VMS often contribute to sleep disturbances and reduced quality of life. Moreover, menopause itself has an increased risk of obstructive sleep apnea (OSA) [34]. GLP-1RAs may provide symptom relief for sleep disturbances; a study conducted by Blackman et al. demonstrated that liraglutide improves a key indicator of OSA severity, apnea-hypopnea index, when compared to placebo [35]. This suggests that GLP-1RAs may address not only weight gain but also sleep-related quality of life concerns in menopausal women.

Potential Cardioprotective and Antioxidant Effects

The apparent increased oxidative stress observed in the post-menopausal state may be mitigated by the implementation of GLP-1RAs. In studies using ovariectomized rat models, treatment with GLP-1RAs was found to induce an antioxidant state. Specifically, liraglutide-treated mice showed increased expression of antioxidant enzymes such as catalase compared with the control group [36]. These findings suggest that GLP-1RAs may help restore some of the cardioprotective effects lost due to estrogen deficiency during menopause. However, further research in human clinical trials is required to fully understand the potential cardioprotective effects of GLP-1RAs in women undergoing the menopausal transition.

Impact on Osteoporosis Development

Osteoporosis is another associated complication of menopause that often requires pharmacological therapy in postmenopausal women. Currently, it is unclear whether GLP-1RAs provide benefits or increase risk for menopausal women. A meta-analysis of randomized controlled trials conducted by Cheng et al. found that liraglutide and lixisenatide reduced the risk for bone fractures in patients with type 2 diabetes (OR: 0.56; 95% CI: 0.38-0.81 and 0.55; 95% CI: 0.31-0.97, respectively), while other GLP-1RA medications such as semaglutide and exenatide showed no improvement (OR: 1.77; 95% CI: 0.87-3.58 and 0.87; 95% CI: 0.39-1.95, respectively) [37]. These findings suggest further research is needed to understand the biochemical processes by which GLP-1RAs modify osteoporosis risk in postmenopausal women.

Discussion

Limitations of the Current Evidence

Many current studies on GLP-1RAs and menopause are preclinical trials in mouse models. While ovariectomized rats are largely regarded as a model for human menopause, they are not the same and have limitations. Therefore, the data from these studies must be analyzed critically. On the other hand, many of the studies conducted in humans were retrospective, limiting the ability to infer true cause-and-effect relationships. Moreover, the human studies cited often have small sample sizes or potential misclassification, which may introduce information bias. Finally, there are no existing long-term studies evaluating the use of GLP-1RAs in the treatment of menopause, as most clinical trials only lasted three to six months.

Cost Barriers of GLP-1RAs

Despite the promising use of GLP-1RAs for symptom management in menopausal women, there remains a significant cost barrier to prescribing and properly administering these medications. Out of pocket, GLP-1RAs may cost upwards of $1000 a month, and many insurance companies only approve prior authorizations if the patient has type 2 diabetes. Moreover, while coverage has recently expanded to include weight loss, many insurance companies require a minimum BMI of 32 kg/m² [38]. Self-pay programs exist for specific medications, such as the Wegovy (semaglutide) direct pay program at $499 a month and Eli Lilly’s LillyDirect program for Zepbound (tirzepatide), with introductory pricing as low as $349 per month [39,40]. Therefore, while GLP-1RAs have the potential to provide significant relief for menopausal women, their use is limited due to cost barriers defined by manufacturers and insurance companies. As such, equitable access must also be established when considering menopausal symptom management as an indication for GLP-1RAs.

Relevance

GLP-1RA medications were initially indicated for type 2 diabetes mellitus and have since been indicated for chronic weight management and in adults with weight-related comorbidities. Given this, future research may also find that menopausal weight gain could be an additional indication of this medication class. While research supports increased weight loss in postmenopausal women using a combination of HT and semaglutide compared with semaglutide alone, it does not claim to improve the symptoms typically experienced during the menopausal transition. Additionally, it does not directly compare the effectiveness of semaglutide and hormonal therapy in treating menopausal symptoms. Given previous research indicating that weight loss can provide symptomatic relief during menopause, there is an opportunity to explore the treatment of menopausal symptoms with GLP-1RAs through weight loss. Only one of the cited studies utilized the menopause quality of life (MENQOL) scale, and it was used to evaluate the relationship between MetS and menopause. This provides an opportunity to research the effects of GLP-1RAs on the patient’s experience of menopause via a standard means of assessing quality of life, the MENQOL scale. Finally, the potential effects of GLP-1RAs on menopausal women may vary based on socioeconomic status, ethnicity, and associated comorbidities.

## Conclusions

This literature review asserts the promising role of GLP-1RAs in the treatment and management of multiple manifestations of menopause. GLP-1RAs are effective for weight management in menopause and may help alleviate symptoms such as VMS. As weight loss itself has been shown to improve quality of life in menopausal women, GLP-1RAs may offer an important therapeutic alternative for patients who remain symptomatic despite treatment with standard care. However, their impact on osteoporosis and CVD risk requires further investigation. Current evidence is limited by reliance on preclinical models and human studies that are retrospective and/or short-term.

Future studies should use tools such as the MENQOL scale to understand the effects of GLP-1RAs on patient quality of life. Moreover, the effects of GLP-1RAs on menopausal women may vary based on socioeconomic status, ethnicity, and associated comorbidities. Therefore, studies should also prioritize diversity to understand the effects of GLP-1RAs in this population.

## References

[1] Christmas M, Janssen I, Joffe H, Upchurch D, Santoro N, Kravitz HM (2022). Menopause hormone therapy and complementary alternative medicine, quality of life, and racial/ethnic differences: the Study of Women's Health Across the Nation (SWAN). Menopause.

[2] Erdélyi A, Pálfi E, Tűű L (2023). The importance of nutrition in menopause and perimenopause-a review. Nutrients.

[3] Woolcott OO, Seuring T (2023). Temporal trends in obesity defined by the relative fat mass (RFM) index among adults in the United States from 1999 to 2020: a population-based study. BMJ Open.

[4] El Khoudary SR, Aggarwal B, Beckie TM (2020). Menopause transition and cardiovascular disease risk: implications for timing of early prevention: a scientific statement from the American Heart Association. Circulation.

[5] Roa-Díaz ZM, Raguindin PF, Bano A, Laine JE, Muka T, Glisic M (2021). Menopause and cardiometabolic diseases: what we (don't) know and why it matters. Maturitas.

[6] Kumar S, Blaha MJ (2024). GLP-1 RA for cardiometabolic risk reduction in obesity - how do we best describe benefit and value?. Am J Prev Cardiol.

[7] Michos ED, Lopez-Jimenez F, Gulati M (2023). Role of glucagon-like peptide-1 receptor agonists in achieving weight loss and improving cardiovascular outcomes in people with overweight and obesity. J Am Heart Assoc.

[8] Davis SR, Castelo-Branco C, Chedraui P, Lumsden MA, Nappi RE, Shah D, Villaseca P (2012). Understanding weight gain at menopause. Climacteric.

[9] Moccia P, Belda-Montesinos R, Monllor-Tormos A, Chedraui P, Cano A (2022). Body weight and fat mass across the menopausal transition: hormonal modulators. Gynecol Endocrinol.

[10] Babaei P, Dastras A, Soltani Tehrani B, Pourali Roudbaneh S (2017). The effect of estrogen replacement therapy on visceral fat, serum glucose, lipid profiles and apelin level in ovariectomized rats. J Menopausal Med.

[11] Shea KL, Gavin KM, Melanson EL (2015). Body composition and bone mineral density after ovarian hormone suppression with or without estradiol treatment. Menopause.

[12] Kanaya AM, Herrington D, Vittinghoff E (2003). Glycemic effects of postmenopausal hormone therapy: the Heart and Estrogen/progestin Replacement Study. A randomized, double-blind, placebo-controlled trial. Ann Intern Med.

[13] Ji MX, Yu Q (2015). Primary osteoporosis in postmenopausal women. Chronic Dis Transl Med.

[14] Llaneza P, González C, Fernandez-Iñarrea J, Alonso A, Arnott I, Ferrer-Barriendos J (2009). Insulin resistence and health-related quality of life in postmenopausal women. Fertil Steril.

[15] Lovre D, Lindsey SH, Mauvais-Jarvis F (2016). Effect of menopausal hormone therapy on components of the metabolic syndrome. Ther Adv Cardiovasc Dis.

[16] Fåhraeus L (1988). The effects of estradiol on blood lipids and lipoproteins in postmenopausal women. Obstet Gynecol.

[17] Kim M, Paik JK, Kang R, Kim SY, Lee SH, Lee JH (2013). Increased oxidative stress in normal-weight postmenopausal women with metabolic syndrome compared with metabolically healthy overweight/obese individuals. Metabolism.

[18] Barrea L, Pugliese G, Laudisio D, Colao A, Savastano S, Muscogiuri G (2021). Mediterranean diet as medical prescription in menopausal women with obesity: a practical guide for nutritionists. Crit Rev Food Sci Nutr.

[19] Kroenke CH, Caan BJ, Stefanick ML (2012). Effects of a dietary intervention and weight change on vasomotor symptoms in the Women's Health Initiative. Menopause.

[20] Berin E, Hammar M, Lindblom H, Lindh-Åstrand L, Rubér M, Spetz Holm AC (2019). Resistance training for hot flushes in postmenopausal women: a randomised controlled trial. Maturitas.

[21] Nilsson S, Henriksson M, Hammar M (2024). A 2-year follow-up to a randomized controlled trial on resistance training in postmenopausal women: vasomotor symptoms, quality of life and cardiovascular risk markers. BMC Womens Health.

[22] Watson SL, Weeks BK, Weis LJ, Harding AT, Horan SA, Beck BR (2018). High-intensity resistance and impact training improves bone mineral density and physical function in postmenopausal women with osteopenia and osteoporosis: the LIFTMOR randomized controlled trial. J Bone Miner Res.

[23] (2014). ACOG Practice Bulletin No. 141: management of menopausal symptoms. Obstet Gynecol.

[24] Walędziak M, Różańska-Walędziak AM (2022). Bariatric surgery and menopause. Prz Menopauzalny.

[25] Andreasen CR, Andersen A, Knop FK, Vilsbøll T (2021). How glucagon-like peptide 1 receptor agonists work. Endocr Connect.

[26] Model JF, Normann RS, Vogt ÉL (2024). Interactions between glucagon like peptide 1 (GLP-1) and estrogens regulates lipid metabolism. Biochem Pharmacol.

[27] Reis-Barbosa PH, Marcondes-de-Castro I, Marinho TS, Aguila MB, Mandarim-de-Lacerda CA (2024). The dual glucose-dependent insulinotropic polypeptide/glucagon-like peptide-1 receptor agonist tirzepatide effects on body weight evolution, adiponectin, insulin and leptin levels in the combination of obesity, type 2 diabetes and menopause in mice. Diabetes Obes Metab.

[28] Park JH, Kim JY, Choi JH (2021). Effectiveness of liraglutide 3 mg for the treatment of obesity in a real-world setting without intensive lifestyle intervention. Int J Obes (Lond).

[29] Nicolau J, Blanco-Anesto J, Bonet A, Félix-Jaume JJ, Gil-Palmer A (2025). Effectiveness of low doses of semaglutide on weight loss and body composition among women in their menopause. Metab Syndr Relat Disord.

[30] (2024). 31st European Congress on Obesity (ECO 2024). Obes Facts.

[31] Tchang BG, Mihai AC, Stefanski A (2025). Body weight reduction in women treated with tirzepatide by reproductive stage: a post hoc analysis from the SURMOUNT program. Obesity (Silver Spring).

[32] Hurtado MD, Tama E, Fansa S (2024). Weight loss response to semaglutide in postmenopausal women with and without hormone therapy use. Menopause.

[33] (2025). Endocrine Society: Combination of obesity medication tirzepatide and menopause hormone therapy fuels weight loss. https://www.endocrine.org/news-and-advocacy/news-room/endo-annual-meeting/endo-2025-press-releases/castaneda-press-release.

[34] Zhou P, Li H, Li H, Chen Y, Lv Y (2025). A possible important regulatory role of estrogen in obstructive sleep apnea hypoventilation syndrome. Front Med (Lausanne).

[35] Blackman A, Foster GD, Zammit G (2016). Effect of liraglutide 3.0 mg in individuals with obesity and moderate or severe obstructive sleep apnea: the SCALE Sleep Apnea randomized clinical trial. Int J Obes (Lond).

[36] Santos WC, Ronchi SN, Gonçalves LA (2025). Liraglutide improves antioxidant defense in hearts of spontaneously hypertensive female rats independently of changes in blood pressure in a pre-clinical model of menopause. Braz J Med Biol Res.

[37] Cheng L, Hu Y, Li YY (2019). Glucagon-like peptide-1 receptor agonists and risk of bone fracture in patients with type 2 diabetes: a meta-analysis of randomized controlled trials. Diabetes Metab Res Rev.

[38] (2025). Glucagon-like peptide-1 receptor agonists: a pharmacy perspective on insurance coverage and medication access. https://wayback.archive-it.org/5818/20250429144950/https://info.primarycare.hms.harvard.edu/perspectives/articles/glp-1-pharmacy-perspective.

[39] INC NN: Novo Nordisk (2025). PR Newswire: Novo Nordisk introduces NovoCare® Pharmacy, lowering cost of all doses of FDA-approved Wegovy® (semaglutide) to $499 per month and offering easy home delivery for cash-paying patients. https://www.prnewswire.com/news-releases/novo-nordisk-introduces-novocare-pharmacy-lowering-cost-of-all-doses-of-fda-approved-wegovy-semaglutide-to-499-per-month-and-offering-easy-home-delivery-for-cash-paying-patients-302392874.html.

[40] Lilly to offer all approved doses of (2025). Lilly: Lilly to offer all approved doses of Zepbound (tirzepatide) single-dose vials through LillyDirect Self Pay Pharmacy Solutions. https://investor.lilly.com/news-releases/news-release-details/lilly-offer-all-approved-doses-zepbound-tirzepatide-single-dose.

